# Interplay of Glucose Metabolism and Hippo Pathway in Chondrocytes: Pathophysiology and Therapeutic Targets

**DOI:** 10.3390/bioengineering11100972

**Published:** 2024-09-27

**Authors:** Jacob Jahn, Quinn T. Ehlen, Lee Kaplan, Thomas M. Best, Zhipeng Meng, Chun-Yuh Huang

**Affiliations:** 1 University of Miami Miller School of Medicine, Miami, FL 33136, USA; tjj57@med.miami.edu (J.J.); qte1@med.miami.edu (Q.T.E.); kaplan@med.miami.edu (L.K.); txb440@med.miami.edu (T.M.B.); zxm282@med.miami.edu (Z.M.); 2 Department of Orthopedics, University of Miami, Miami, FL 33136, USA; 3 UHealth Sports Medicine Institute, University of Miami, Miami, FL 33136, USA; 4 Department of Molecular and Cellular Pharmacology, Miller School of Medicine, Miami, FL 33136, USA; 5 Sylvester Comprehensive Cancer Center, University of Miami Miller School of Medicine, Miami, FL 33136, USA; 6 Department of Biomedical Engineering, University of Miami, Coral Gables, FL 33146, USA

**Keywords:** glucose, metabolism, mechanotransduction, chondrocyte, osteoarthritis, OA therapeutics

## Abstract

In this review, we explore the intricate relationship between glucose metabolism and mechanotransduction pathways, with a specific focus on the role of the Hippo signaling pathway in chondrocyte pathophysiology. Glucose metabolism is a vital element in maintaining proper chondrocyte function, but it has also been implicated in the pathogenesis of osteoarthritis (OA) via the induction of pro-inflammatory signaling pathways and the establishment of an intracellular environment conducive to OA. Alternatively, mechanotransduction pathways such as the Hippo pathway possess the capacity to respond to mechanical stimuli and have an integral role in maintaining chondrocyte homeostasis. However, these mechanotransduction pathways can be dysregulated and potentially contribute to the progression of OA. We discussed how alterations in glucose levels may modulate the Hippo pathway components via a variety of mechanisms. Characterizing the interaction between glucose metabolism and the Hippo pathway highlights the necessity of balancing both metabolic and mechanical signaling to maintain chondrocyte health and optimal functionality. Furthermore, this review demonstrates the scarcity of the literature on the relationship between glucose metabolism and mechanotransduction and provides a summary of current research dedicated to this specific area of study. Ultimately, increased research into this topic may elucidate novel mechanisms and relationships integrating mechanotransduction and glucose metabolism. Through this review we hope to inspire future research into this topic to develop innovative treatments for addressing the clinical challenges of OA.

## 1. Introduction

### Chondrocyte Physiology

Chondrocytes, the predominant cell within articular cartilage (AC), play an important role in the maintenance of joint integrity, and in the prevention of cartilage-derived pathologies, such as osteoarthritis (OA) [1]. Their core function is achieved through the maintenance of a robust network of extracellular matrix (ECM) consisting of collagen and proteoglycans. Importantly, the ECM of AC serves to dissipate mechanical stresses due to its uniquely resistant nature, ensuring shock absorption and high levels of resilience to mechanical loading [2,3].

OA, widely characterized as a degenerative/inflammatory joint disease stemming from the degeneration of AC and underlying bone, imposes a costly burden on the healthcare system [4,5]. Risk factors including aging, obesity, prior joint injury, and recurrent and persistent joint stress were explored rigorously to identify potential therapeutic means for lessening or eliminating the detrimental effects of OA [2,6,7,8,9]. Furthermore, OA was identified as one of the most prevalent chronic diseases worldwide, with recent studies estimating a global prevalence of 595 million, a remarkable 132.2% increase since 1990 [4,10]. Patients with OA suffer from vastly increased chronic pain, diminished mobility, and overall functional impairment [11,12,13], which contributes to the formidable challenge presented by OA management. To date, investigations into the underlying pathophysiology of OA have postulated that an imbalance between the anabolic and catabolic activity of chondrocytes leads to an inability to replenish and maintain healthy AC [14,15,16,17]. This, coupled with additional changes in subchondral bone and inflammatory activities of synovium cells, are ultimately responsible for the clinical picture of OA [18,19].

Chondrocytes promote AC and joint integrity through the generation of ECM products, specifically collagen and proteoglycans, which provide structural support to AC. Chondrocytes are widely dispersed throughout the body; however, they are highly concentrated in areas exposed to increased mechanical stress or loading [20,21] which results in a highly specialized makeup of collagenous elements providing tensile strength and proteoglycan network imparting an inherent compressive resistance and elasticity [20,22]. Additionally, chondrocytes maintain a fine-tuned balance of metabolic regulation within AC, ensuring that both anabolic and catabolic activities, responsible for matrix synthesis and destruction, respectively, are maintained within a range suitable to manage the biomechanical demands of AC [23,24].

While chondrocytes possess the capacity to perform fundamental functions such as the generation of cartilaginous matrix, it is important to note that their distribution and structural arrangement varies based upon the local mechanical demands [14,25]. For instance, chondrocytes are distributed in several distinct zones within AC: superficial, middle, and deep [23]. Within each of these zones, chondrocytes possess unique cellular arrangements and morphological characteristics that optimize their ability to meet local functional demands [20,23]. Additionally, throughout unique anatomical locations in the body, such as AC and intervertebral discs (IVD), their biomechanical functions vary slightly [20,26]. For example, chondrocytes located within the AC have the primary function of bearing compressive forces [27], while in the IVD, they facilitate load bearing while simultaneously meeting the flexibility requirements of the spine [26].

Therefore, it is evident that chondrocytes exhibit tissue-specific variations in their physiology and functionality, dependent on the mechanical demands present in their particular anatomical locations. Similarly, chondrocytes demonstrate location-dependent differences in metabolic activity and response to mechanical stress. Despite these minute differences, the overarching issue is the catabolic activities of chondrocytes enhanced by pathological mechanical conditions, which leads to a failure to maintain a regulatory environment in the surrounding tissue, irrespective of its anatomical location. Thus, it is imperative to investigate and identify targetable elements within the mechanosignaling pathways affecting chondrocyte metabolism to develop effective therapeutic strategies [2,28].

Within this review, we seek to comprehensively examine two specific elements that are integral to chondrocyte metabolism and overall functioning: mechanical loading, which induces various intricate and largely uncharacterized mechanotransduction pathways [9,28], and glucose metabolism, which, as in the vast majority of biochemical metabolic pathways, is responsible for the propagation and execution of cellular functionality [29]. Through characterization of the roles of mechanotransduction and glucose metabolism on chondrocyte function, we also aim to explore the interplay and crosstalk of these vital elements, to identify a relationship that can potentially be leveraged in the management of OA and other cartilage-related diseases.

## 2. The Role of Mechanotransduction Pathways in Chondrocyte Metabolism

### 2.1. Introduction to Mechanotransduction

Chondrocytes, like many cell types, utilize various mechanoreceptors to detect and effectuate responses to mechanical stimuli [28]. In order to achieve this function, there are several categories of mechanoreceptors, such as integrins, ion channels, and cytoskeletal elements, like primary cilia, which ultimately translate physical forces into biochemical signaling pathways to initiate a cellular metabolic response [28,30,31]. These metabolic responses facilitate the proliferation and differentiation of chondrocytes and account for the previously discussed tissue-specific functional specialization in high-impact regions of the body. This high degree of fidelity is essential to tissue-organ homeostasis, as mechanotransduction and its diverse intermediates allow for the adaptation of cellular behavior via alterations in gene expression, protein synthesis, and cellular structure organization to coordinate an optimal response to imposed mechanical changes [32,33].

To receive and facilitate the propagation of mechanical signals, chondrocytes possess several mechanosignaling cascades, including those associated with Mitogen-Activated Protein Kinase (MAPK), Phosphoinositide 3-Kinase (PI3K), protein kinase C (PKC), and Yes-Associated Protein/Transcriptional Co-activator with PDZ-binding Motif (YAP/TAZ), and others [28,34]. In a system resembling that of electrical wiring or a line of dominoes, mechanical stimuli are carried forth via phosphorylation events, which influence chondrocyte metabolism. As described in our previous review [9], an optimal range of mechanical loading is necessary to activate mechanotransduction pathways to support cartilage anabolism.

As discussed previously, mechanical conditions play an integral role in the promotion of anabolic and catabolic activities of chondrocytes. Based on the review by Sanchez-Adams et al. [2], it was seen that moderate mechanical loading promotes anabolic activities, such as cartilage matrix synthesis and the maintenance of tissue homeostasis. Conversely, excessive or insufficient loading contributes to catabolic activities, ultimately resulting in AC degradation and initiation or progression of OA [2]. The mechanosignaling cascades are responsible for adapting the regulatory response to supra-threshold or insufficient mechanical loading. This process is typically achieved by triggering apoptosis using intracellular pathways incorporating p53 and caspases, to maintain tissue homeostasis through the removal of damaged-beyond-repair cells [35,36]. Yet, within these catabolic pathways in OA pathogenesis, enzymes like matrix metalloproteinases (MMPs) and disintegrin and metalloproteinases (ADAMs) have been demonstrated to be upregulated following bouts of excessive loading [37,38]; consequently, there is degradation of ECM and weakening the structure and functionality of chondrocytes, culminating in programmed cell death. Similarly, during prolonged periods of sub-threshold mechanical loading, anabolic signaling may be decreased, leading to decreased chondrocyte ECM synthesis, chondrocyte death, cartilage thinning, and ultimately progression of OA [39,40].

To explore the role of mechanotransduction in OA, and its relationship to glucose metabolism in OA pathogenesis, it is essential to carefully introduce key mechanotransduction pathways proven to play a role in chondrocyte metabolism. Next, we will discuss the involvement of integrins and ion channels in mechanotransduction and their role in initiating chondrocyte response to mechanical loading. From this, we will delve deeper into the burgeoning research surrounding the Hippo pathway to provide the foundation for a link between mechanotransduction pathways and glucose metabolism.

### 2.2. Integrins

In AC, the interaction between chondrocytes and ECM is facilitated by essential transmembrane receptors known as integrins. The underlying mechanism of this chondrocyte–ECM interaction is complex, but it ultimately surrounds the binding of integrins to ECM proteins such as fibronectin and collagen leading to the formation of focal adhesion complexes [30]. When these complexes are formed and activated, kinases such as Focal Adhesion Kinase (FAK) and SRC are recruited and stimulated, leading to downstream signaling in mechanotransduction pathways [33]. As mentioned previously, the downstream mechanotransduction pathways maintain a proper balance of cytoskeletal organization, gene expression, and cell survival, which contribute to the maintenance of proper cartilage structure and function and response to mechanical forces [41]. Accordingly, integrins play a pivotal role in chondrocyte mechanotransduction through the conversion of external mechanical stimuli into intracellular responses that can facilitate remodeling and other chondrocyte events.

### 2.3. Ion Channels

Similar to integrins, ion channels, especially those belonging to the transient receptor potential (TRP) family, are key elements in chondrocyte mechanotransduction [42]. In response to mechanical forces like chronic compression or osmotic changes following acute injury, the conformation of TRP channels is altered, leading to an influx of Ca^2+^ ions across chondrocyte cell membranes [43,44,45]. In many metabolic pathways, Ca^2+^ is an important activator of calcium-dependent intracellular signaling pathways that contribute to the chondrocyte’s response to mechanical stimulation [46]. Additionally, TRP channels were shown to modulate inflammatory responses and matrix synthesis [47], which, as mentioned, are important in the maintenance of overall cartilage health. In short, through their role as mechanosensors, TRP channels help facilitate the precise intra-chondrocyte environment necessary to allow for proper functioning.

### 2.4. Primary Cilia

Primary cilia, microtubule-based organelles present on the surface of most mammalian cells, including chondrocytes, play an important role in mechanotransduction [48]. In order to accomplish this, primary cilia act as sensory hubs that are capable of detecting and responding to various mechanical and chemical sensory inputs present in the extracellular environment [49]. Hodgkinson et al. have highlighted the critical function of primary cilia in cartilage mechanosignaling, with a specific focus on OA [28]. Importantly, they conclude that primary cilia act as a convergence point for several mechanotransduction pathways, such as Hedgehog, Wnt, and platelet-derived growth factor (PDGF) signaling, all of which are implicated in chondrocyte biological processes [28]. Mechanistically, primary cilia are responsible for the transduction and conversion of chemical and mechanical signals into intracellular biochemical responses through the activation of ion channels and integrins on the chondrocyte membrane [50]. The Polycystin complexes (PC1 and PC2) are one of the few critical mechanosensitive receptors present on primary cilia, which mediate calcium influx and propagate downstream signaling in response to mechanical stimuli [50,51]. Additionally, investigations into primary cilia indicate that they may influence a cell’s response to inflammatory cytokines, such as Interleukin 1β (IL-1β) and tumor necrosis factor α (TNF-α) [52]. These inflammatory markers and cellular responses may alter downstream signalizing pathways and exacerbate cartilage degeneration during a host inflammatory state.

### 2.5. Hippo Pathway

**Figure 1:**
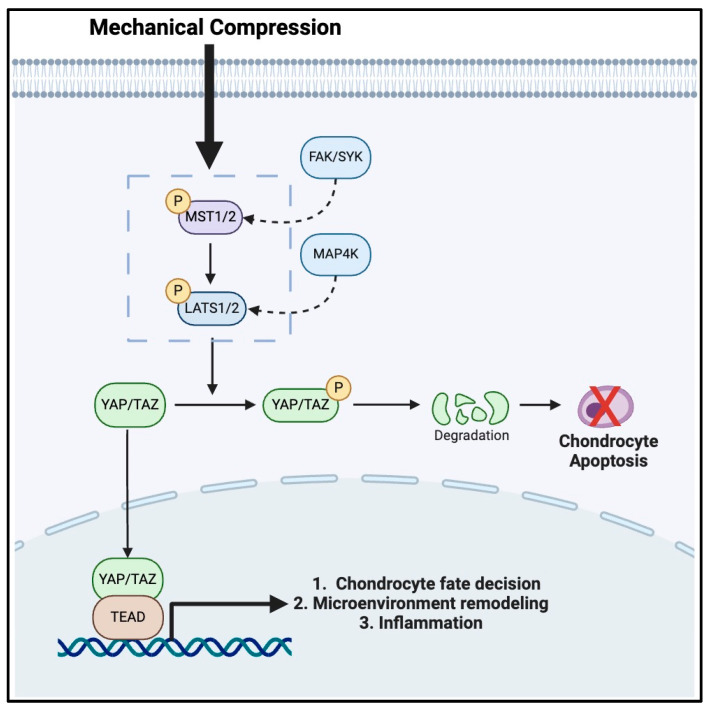
The mechanotransduction pathway in chondrocytes mediated by the HIPPO signaling cascade.

The Hippo pathway is a well-established regulator of inflammation and chondrocyte mechanotransduction and involves several key molecular players including YAP/TAZ, Mammalian Ste20-like Kinase1/2 (MST1/2), and Large Tumor Suppressor1/2 (LATS1/2) [53,54,55,56] (Figure 1). These kinase proteins are responsible for the translation of mechanical stimuli detected by integrins and ion channels into intracellular signals responsible for physiologic responses [54]. Similar to the analogy we discussed earlier, this pathway acts like unidirectional electrical wiring. To ensure proper functioning, MST1/2 is phosphorylated by upstream signals, such as FAK and Syc [34], as mentioned in the previous section on integrins. Once activated, MST1/2 then phosphorylates LATS1/2, which subsequently goes on to phosphorylate YAP/TAZ linearly [54]. However, the effectuation of this pathway is more complex, as once YAP/TAZ is phosphorylated, it is retained in the cytoplasm and ultimately degraded [54]. However, during periods of mechanical loading or stretching, LATS1/2 phosphorylation may be inhibited, thus promoting the activation of YAP/TAZ. This allows YAP/TAZ to migrate into the nucleus of chondrocytes where they bind to a family of transcription factors called the TEA Domain (TEAD), which enhances chondrocyte proliferation and survival and also promotes ECM development [57,58,59]. Thus, it is evident that mechanical stress regulates chondrocyte functions via the Hippo pathway, as YAP/TAZ nuclear localization promotes chondrocyte-maintaining events that support cartilage growth and repair [60]. Conversely, LATS1/2-mediated phosphorylation of YAP/TAZ can induce chondrocyte apoptosis via the cytoplasmic sequestration of YAP/TAZ, which may occur when chondrocyte proliferation may not be necessary [60].

Furthermore, the Hippo pathway plays a significant role in both pro- and anti-inflammatory disease states [61,62,63,64], especially involving YAP. Primarily, YAP activation can lead to the production of pro-inflammatory cytokines, which may accelerate and contribute to cartilage degradation in OA [65,66,67,68]. In contrast, YAP activation can also promote an anti-inflammatory response leading to tissue repair and regeneration following an inflammation-inducing event, such as acute injury [67]. Importantly, studies have demonstrated that inflammation can activate YAP, contributing to its nuclear localization and pro-inflammatory gene expression thereby leading to exacerbation of clinical symptoms of OA [66].

As reviewed previously, it is evident that the relationships between the Hippo pathway kinases are largely responsible for physiologic tissue homeostasis. However, it should also be noted that when the Hippo pathway is disrupted or dysregulated due to abnormal or excessive mechanical loading, detrimental effects, such as impaired chondrocyte proliferation or increased chondrocyte death, may occur and threaten the fine-tuned balance necessary to maintain proper chondrocyte and cartilage function [28,69,70,71]. By understanding the intricate details of the Hippo pathway, it is clear that this pathway may be a source of potential development of targeted therapies addressing underlying chondrocyte pathology [72]. By targeting specific molecules within the Hippo pathway, such as enhancing YAP/TAZ nuclear sequestration or inhibiting excessive LATS1/2 activity, potential treatments could mitigate the progression of OA. Additionally, this section provides a bridge to our expanded discussion on the crosstalk of glucose metabolism and mechanotransduction in chondrocytes, as there is growing evidence that glucose metabolism interacts closely with the Hippo pathway via YAP/TAZ activity, which may modulate the sensitivity of chondrocytes to mechanical loading [73,74,75].

## 3. The Role of Glucose Metabolism in Inflammatory Responses of the Joints

Traditionally, excessive mechanical load (traumatic or atraumatic) and aging have been the frontrunners of etiologies leading to OA initiation and progression. It was demonstrated that individuals with obesity place larger loads on their joints, leading to a pro-inflammatory state and degradation of the joints, most commonly in the weight-bearing joints. The pro-inflammatory state in individuals with obesity is characterized by increased levels of IL-1β, Interleukin 6 (IL-6), and TNF-α, which have been implicated in the pathophysiology of OA [76,77,78]. However, individuals with metabolic disease also have an increased incidence of OA in non-weight-bearing joints, leading to investigations into the underlying mechanism contributing to the metabolic effects [79]. Originally, it was thought that the correlation between obesity and diabetes mellitus (DM) explained why many individuals with DM develop OA; however, more recent studies suggest a pro-inflammatory mechanism of hyperglycemia and DM that may contribute to OA development [80]. Freemerman et al. demonstrated that excessive glucose metabolism promotes a hyperinflammatory state via increased pentose phosphate intermediates and macrophage activity [80]. Hyperglycemia also increases glycolysis, which was also shown to produce a pro-inflammatory state [81]. These findings have sparked a new effort to understand how glucose metabolism affects chondrocyte activity in the development of OA.

### Potential Mechanisms of Hyperglycemia-Induced Inflammatory Responses of the Joints

**Figure 2:**
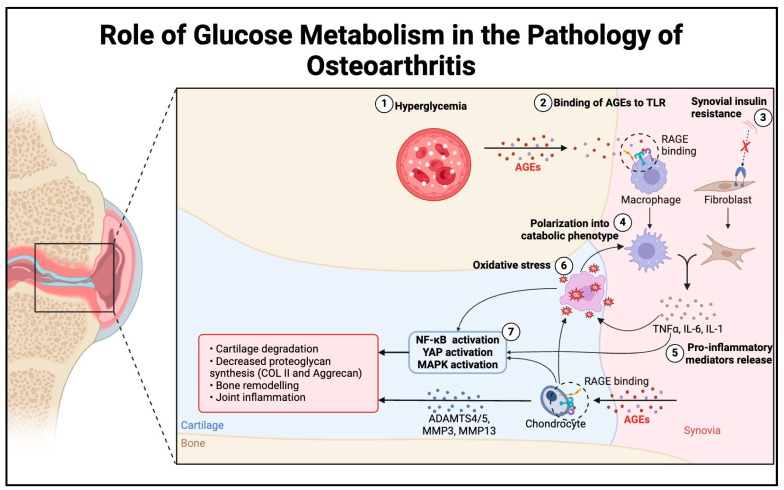
The schematic of the proposed mechanism of increased sensitivity to inflammatory responses in hyperglycemic conditions. Hyperglycemia results in higher concentrations of AGEs, leading to increased RAGE binding on macrophages and chondrocytes. Meanwhile, chronic hyperglycemia leads to synovial insulin resistance. Both sequences induce polarization of macrophages and fibroblasts into catabolic phenotypes, which increase inflammatory mediators and cytokines. The inflammatory response from macrophages and fibroblasts, in combination with a chondrocyte response, activates NF-κB and MAPK, increases oxidative stress, and creates a positive inflammatory feedback loop leading to cartilage degradation and OA progression.

The effect of glucose metabolism on chondrocytes has been extensively studied. It is known that elevated blood glucose incites a pro-inflammatory response within chondrocytes. Chronic hyperglycemia leads to advanced glycation end products (AGEs) which bind to the receptor for AGEs (RAGE), stimulating multiple intracellular pathways [82,83]. Overall, binding to RAGE leads to a pro-inflammatory state via Nuclear Factor kappa-light-chain-enhancer of activated B cells (NF-κB) and MAPK activation, leading to Interferon (IFN)-gamma and IL-6 responses [84]. Additionally, AGEs also affect the ECM by impeding its turnover. Ultimately, the culmination of these inflammatory responses leads to increased degradation and diminished proteoglycan synthesis, resulting in unhealthy cartilage [85]. Therefore, hyperglycemia could induce a pro-inflammatory state that could contribute to OA development and progression (Figure 2).

Our understanding of the biochemical effects of hyperglycemia on OA was further confirmed using in vivo models. Mouse models have demonstrated that animals fed high-fat diets demonstrated an increased pro-inflammatory state through increased levels of IL-1β and IL-6 [86,87]. In another study by Li et al., it was found that a pro-inflammatory state with excess IL-1β and TNF- α led to increased expression of MMP3, MMP13, and ADAMTS4, all of which induce a catabolic response within AC [88]. The biochemical catabolism results in morphological changes, including synovial inflammation and subchondral bone thickening [19,89]. Supplementation with metformin was shown to protect against OA through inhibition of the inflammatory response via reduced blood glucose and decreased expression of MMP3 and MMP13 [88]. The protective effects of metformin on OA have also been replicated in humans with DM [90,91,92].

Aside from hyperglycemia inducing a host of inflammatory responses, DM also contributes to insulin resistance, which is theorized to accelerate OA via a separate mechanism. In healthy patients’ synovium, insulin inhibits the expression of TNF-α, TNF-mediated cytokines, and proteases [93]. However, due to chronic hyperglycemia, individuals with DM develop insulin resistance. As a result, their insulin receptors become less functional, leading to decreased inhibition of TNF and its concurrent inflammatory response [94]. Overall, insulin resistance, combined with increased AGEs and oxidative stress, is associated with OA progression and places those with DM at high risk relative to the non-diabetic population [94].

These pathophysiological findings of DM’s effects on OA give rise to many clinical considerations in understanding the risk of OA development. The etiology of OA is multifactorial, a combination of mechanical and biochemical forces that ultimately lead to cartilage destruction. While obesity and its mechanical effects are often considered in this context, other factors of metabolic diseases must not be overlooked. In a cross-sectional study conducted by So et al., a significant positive association in women was found between a high glycemic index and symptomatic knee OA, when adjusting for factors such as age, physical activity, and obesity [95]. Other studies have alluded to various diets having a lower prevalence of OA, suggesting that metabolism plays a significant role in the pathogenesis of the disease [96,97,98]. Because there are strong relationships between metabolic disorders, such as obesity and DM, individuals with multiple co-morbidities are at a synergistically high risk of OA progression. Tight glycemic control is closely related to the prevention of OA progression. Using lifestyle changes, anti-hyperglycemic medications, and newer options like Glucagon-Like Peptide 1 (GLP-1) agonists, providers aim to keep hemoglobin A1c less than 7% to minimize the development of AGEs and decrease the speed of insulin resistance [99]. Additionally, the only curative treatment to date for those experiencing OA is total joint replacement. While weight loss, glucose control, physical therapy, and various injections temporize the symptoms, total joint replacement remains the only curative treatment option. While it varies from surgeon to surgeon, orthopedic surgeons often require a hemoglobin A1c below 8% due to the negative effects of poor glycemic control on wound healing [100,101,102,103]. This is yet another example of the importance of glycemic control for those with diabetes and OA—to prevent further OA progression and to have the opportunity to undergo curative treatment, should the disease progress to that point.

## 4. Interplay between Mechanotransduction and Glucose Metabolism

Based on our discussion thus far, covering both mechanotransduction pathways and glucose metabolism, it is evident that each of these elements contributes strongly to the pathogenesis of OA. However, what is less clear, and vastly uncharacterized in existing literature, is the detailed interplay of mechanotransduction and glucose metabolism, which necessitates further exploration into potential linkages between these contributory factors in the broader context of OA pathophysiology.

In the preceding section, we review the role of the glucose metabolic cascade in OA, with a particular focus on how glucose levels determine inflammatory responses conducive to OA progression. However, glucose signaling and energy availability are also critical elements in homeostasis and physiologic cellular events, as these influence cellular responses and provide the capacity for the fulfillment of desired cell functions [104,105]. In previous investigations [106,107,108], it was shown that mechanical signals influence glucose metabolism by altering cellular energy demand and utilization of substrates. To effectuate this response, increased mechanical loading can enhance the activities of glucose transporters and glycolytic enzymes to accommodate for an increased need to respond to mechanical events [109,110]. Conversely, glucose availability can, in turn, modulate the responsiveness of cells to mechanical stress [29]. In chondrocytes specifically, it is postulated that high glucose concentrations enhance their capacity to sense and respond to mechanical stimuli, as there is an increased ability of intermediary kinases to signal their specific downstream effectors in response to mechanical loading [111,112]. Ultimately, the influences of glucose create a feedback loop in which chondrocytes modulate their metabolic response to mechanical stimuli to maintain homeostasis and support cartilage integrity and function [74].

However, the recent literature has implicated glucose levels as a potential driver of adverse chondrocyte responses to mechanical stimuli [29]. With regard to the feedback loop described above, hyperglycemia and aberrant glucose metabolism may disrupt the delicate balance between mechanotransduction and glucose signaling [64,113]. Consequently, this pro-inflammatory state can impair the mechanosensitive response of chondrocytes, leading to decreased signaling, inhibition of chondrocyte function, and progression of OA [114]. In this section, we seek to explore potential mechanisms by which this pathogenic process occurs, exploring the interplay of glucose metabolism and mechanotransduction and their synergistic effects on the Hippo pathway, to identify potential targets for therapeutic intervention in OA pathogenesis.

### Influence of Glucose on the Hippo Pathway

Glucose metabolism may influence specific Hippo intermediates that are dependent on and sensitive to intracellular glucose concentration [75]. For example, high concentrations of glucose induce the PI3K/Akt pathway [115], which affects the phosphorylation status and activity of MST1/2 and LATS1/2. These integral regulators determine the final nuclear localization of YAP/TAZ within the Hippo pathway [34,54].

In addition to the activation of auxiliary pathways that interact with the Hippo pathway, glucose levels also impact metabolic sensors that induce the Hippo mechanotransduction cascade. Amongst the metabolic sensors are AMP-activated protein kinase (AMPK) and mTOR, which influence the cellular localization of YAP/TAZ [116,117,118,119]. More specifically, investigations by Mo et al. have demonstrated that under low energy conditions, where glucose concentration is sub-optimal, AMPK inhibits YAP/TAZ phosphorylation and nuclear migration, rendering it trapped in its inactive conformation within the cytoplasm of chondrocytes [116,120]. This inhibitory action ensures that during times of limited energy supply, cell proliferation is curtailed. However, following acute knee injury as in post-traumatic OA (PTOA) or chronic OA progression, having concomitant low glucose concentration may contribute to chondrocyte failure of self-renewal, loss of cartilage regeneration, and OA progression [29]. Alternatively, mTOR, which is activated by glucose abundance, enhances YAP/TAZ activity via the facilitation of its nuclear migration [121]. Under periods of proper glucose signaling, the role of mTOR signaling is beneficial, as it allows for chondrocyte proliferation and maintenance of cartilage structure [122]. However, under hyperglycemic conditions in which mTOR activity is irregularly activated, excess chondrocyte proliferation may induce abnormal cartilage growth, leading to the development of osteophytes, a hallmark of OA [123]. Furthermore, hyperglycemic conditions may also contribute to OA development through glucose-dependent activation of YAP. Research shows that glucose can regulate Hippo signaling by specifically enhancing YAP activity [75], which was linked to OA progression [69,70,71]. Increased glucose supply promotes glycolysis, which suppresses AMPK’s inhibition of YAP by lowering the AMP:ATP ratio and increasing the glycolytic intermediate fructose-1,6-bisphosphate [75,119]. Elevated glycolysis also enhances uridine diphosphate N-acetyl glucosamine (UDP-GlcNAc) production via the hexosamine biosynthesis pathway, which activates YAP through O-GlcNAcylation [75,124]. Additionally, higher glucose levels trigger insulin release, activating a cascade involving PI3K, PDK1, and AKT, which prevents YAP degradation through a kinase relay (MST1/2-LATS1/2) [75,119]. Given that excessive YAP activation has been associated with OA development [69,70,71], metabolic abnormalities such as DM may be significant risk factors for OA.

Aside from its effects on Hippo pathway effector kinases (e.g., mTOR and AMPK), hyperglycemia also influences the Hippo pathway through its modulation of the activity of the mechanosensitive channels and integrins discussed previously [125]. Increased glucose concentrations enhance glycolytic flux, which increases the production of metabolic intermediates such as pyruvate and lactate, which directly interact with the Hippo pathway [126]. The upregulation of glycolytic activity experienced during hyperglycemic events may alter the sensitivity and responsiveness of chondrocytes to mechanical stimuli, impairing their target function. Therefore, both hypo- and hyperglycemic conditions possess the capability to interrupt proper Hippo signaling and mechanotransduction through the enhancement of glycolytic flux, suppression of AMPK, or the over-activation of mTOR signaling. These effects ultimately alter YAP/TAZ activity, leading to apoptosis and inadequate chondrocyte regeneration in hypoglycemic conditions, or uncontrolled, unorganized chondrocyte proliferation in periods of hyperglycemic conditions. Much like the “Goldilocks” zone described with regard to proper mechanical loading [9], it is evident that the maintenance of glucose concentrations within an optimal range is critical to proper chondrocyte function. Studies using diabetic models within OA research have demonstrated this phenomenon, as studies have demonstrated that glucose levels that are too low, or too high, disrupt proper Hippo signaling, ultimately leading to dysregulated chondrocyte growth and OA progression [29].

Finally, a potential mechanism for glucose influencing cartilage degradation and OA progression can be seen in the formation of AGEs under hyperglycemic conditions [127]. AGEs, as mentioned previously, interfere with the proper signaling of various pathways, including Hippo [128]. Within Hippo, we postulate that AGEs alter intra-cascade kinases and receptors involved in the propagation of mechanosignaling via the activation of pro-inflammatory pathways, such as NF-κB and MAPK [129,130], which further underscores the detrimental effects of chronically high glucose levels on proper cartilage health.

**Figure 3:**
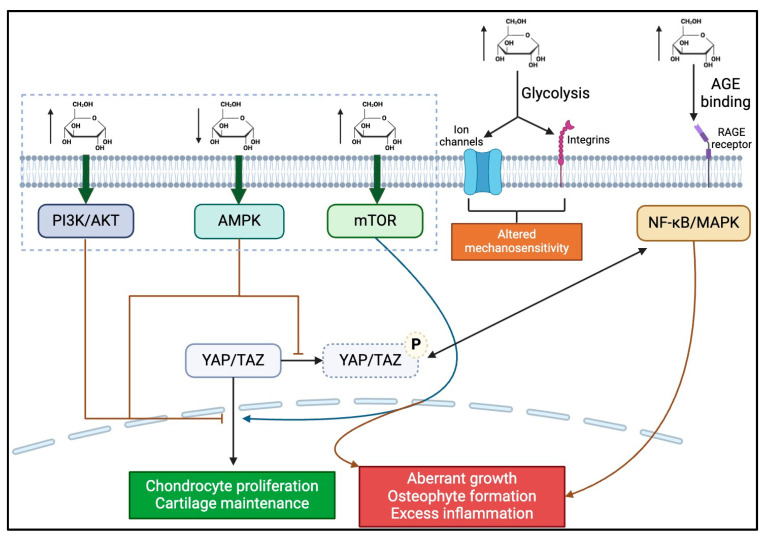
The schematic of the proposed biochemical mechanism by which hyperglycemia may contribute to OA progression. Hyperglycemic conditions lead to the activation of PI3K, AKT, mTOR, NF-κB, and MAPK pathways, which have various intracellular effects, most of which lead to OA progression (as indicated by red arrows/inhibitors). Conversely, hypoglycemic conditions activate the AMPK pathway, also leading to OA progression. Increased blood glucose also leads to increased glycolysis, which alters the mechanosensitivity of chondrocytes by changing ion channels and integrin activity. Finally, hyperglycemia also leads to increased ROS and RAGE binding, leading to a pro-inflammatory state.

Ultimately, these fine-tuned interactions of intracellular metabolites highlight the importance of maintaining balanced metabolic and mechanical signals to maintain proper Hippo activation, chondrocyte activity, and joint health. Abnormal glucose levels may lead to the formation of AGEs to create a perpetual cycle of cellular stress and an inflammatory environment, which threatens the optimized function of chondrocytes and contributes to the pathogenesis of OA (Figure 3). Heretofore, there are limited investigations utilizing in vitro and in vivo models to characterize these elements, particularly about the crosstalk of mechanotransduction and glucose metabolism in OA (Table 1). The following are several recommended research directions to further explore the role of glucose metabolism in mechanical load-induced OA:

Targeting metabolic pathways: Future studies should investigate the interaction between glucose metabolism and Hippo signaling in the development of mechanical load-induced OA. Understanding how glucose metabolism contributes to this form of OA may lead to therapies that address both metabolic and mechanical factors of the disease.

Biomarkers of glucose-enhanced cartilage damage: Identifying biomarkers associated with glucose-driven cartilage damage from mechanical stress could enable early detection and intervention, particularly for patients with metabolic disorders.

Therapeutic interventions: Research into glucose-lowering and anti-inflammatory therapies in OA models with metabolic dysfunction could reveal whether managing glucose levels can reduce adverse mechanotransduction effects.

Advanced OA models: There is a need for more sophisticated in vitro and in vivo OA models that mimic both metabolic changes and mechanical stress in cartilage. Such models will help clarify the balance between glucose levels, mechanotransduction, and cartilage homeostasis.

Exploring glucose’s role in other joint tissues: While cartilage has been a major focus, other joint tissues, such as the synovium and subchondral bone, are also influenced by mechanical stress and glucose levels. Investigating glucose’s impact on these tissues could expand OA research.

## 5. Conclusions

In this review, we sought to investigate potential linkages between underlying chondrocyte metabolism and mechanotransduction pathways, with particular emphasis on the Hippo signaling pathway. Furthermore, we discussed the impact of glucose metabolism on chondrocyte function and its potential role in the progression of OA. Importantly, through further characterization of mechanotransduction and glucose metabolism, and evaluation of their interplay, we seek to highlight the promising therapeutic potential of targeting these pathways in OA pathogenesis.

Despite the topics raised within this review, the current literature evaluating the crosstalk between glucose metabolism and Hippo signaling using in vitro and in vivo models is somewhat limited. Ultimately, this gap represents an area of current investigation by our group, which is focused on identifying the molecular mechanisms underlying the interplay between glucose and mechanical transduction and their synergistic roles in OA progression, in order to be harnessed for future OA therapy.

## Figures and Tables

**Table 1:** Literature on mechanotransduction and glucose metabolism in chondrocytes.

Study Focus	Author, Year	Study Type	Study Topic	Source
**Mechanotransduction in Chondrocytes**	Goldring, 2000	Review	Overview of chondrocyte function in OA	[1]
	Findlay, 2014	Review	Interaction of chondrocytes and osteoblasts in OA	[131]
	Hodgkinson, 2022	Review	Mechanosignaling as a treatment for OA	[28]
	Gilbert, 2018	Review	Response of chondrocytes to mechanical load	[132]
	Jahn, 2024	Review	Optimal mechanical loading for treatment and prevention of OA	[9]
	Zhang, 2024	Review	Mechanobiology of chondrocytes	[133]
	Dieterle, 2021	Review	Role of integrins and channels in cartilage mechanotransduction	[30]
	Gao, 2022	Review	Mechanosensitive channels in OA pathogenesis	[134]
	Shioji, 2014	**In vitro**	Intracellular mechanisms of mechanotransduction in OA	[135]
**Glucose Metabolism in Chondrocytes**	Freemerman, 2014	**In vivo**	Inflammatory effects of GLUT-1 mediated glucose metabolism	[80]
	Puleston, 2017	Review	Role of glucose metabolism in inflammation	[81]
	Li, 1997	**In vitro**	Analysis of RAGE and its role in glucose metabolism	[84]
	Rendra, 2019	Review	Role of ROS in diabetes and glucose metabolism	[89]
	Pi, 2024	Review	Role of intermediates in chondrocyte glucose metabolism	[29]
	Defois, 2023	**In vitro**	Glucose metabolism in OA chondrocytes	[136]
**Interaction of Mechanotransduction and Glucose Metabolism in Chondrocytes**	Jørgensen, 2017	Review	Effects of mechanical loading on glucose metabolism	[82]
	Romani, 2021	Review	Crosstalk of mechanotransduction and glucose metabolism	[74]
	Solinas, 2022	Review	Role of PI3K/Akt mechanotransduction signaling and metabolism	[115]
	Hollander, 2019	Review	Role of glucose metabolism in chondrocyte development	[113]
	Zheng, 2020	Review	Interaction of Hippo signaling and metabolism	[137]
	Ibar, 2020	Review	Integration of Hippo and metabolism	[75]
	Salinas, 2017	**Review**	Role of mechanotransduction on glucose metabolism	[138]
	Zignego, 2015	**Review**	Role of glucose metabolism on mechanotransduction pathways	[139]

## Data Availability

Not applicable.

## Abbreviations

ADAMs: A disintegrin and metalloproteinases; AGEs: Advanced Glycation Endproducts; AMPK: AMP-activated protein kinase; ATP: Adenosine Triphosphate; Ca^2+^: Calcium ionDM: Diabetes Mellitus; ECM: Extracellular Matrix; FAK: Focal Adhesion Kinase; GLP-1: Glucagon-Like-Peptide-1; GLUT-1: Glucose Transporter 1; HIPPO: Hpo; IFN-gamma: Interferon gamma; IGF-1: Insulin-like Growth Factor 1; IL-1: Interleukin 1; IL-6 Interleukin 6; IL-1β: Interleukin 1 beta; IVD: Intervertebral Discs; LATS1/2: Large Tumor Suppressor ½; MAPK: Mitogen-Activated Protein Kinase; =MMPs: Matrix Metalloproteinases; MST1/2: Mammalian Ste20-like Kinase ½; NFκB: Nuclear Factor kappa-light-chain-enhancer of activated B cells; OA: Osteoarthritis; PC1/2: Polycystin 1/2 Complexes; PDGF: Platelet-derived Growth Factor; PI3K: Phosphoinositide 3-Kinase; PTOA: Post-traumatic osteoarthritis; ROS: Reactive Oxygen Species; RAGE: Receptor for Advanced Glycation Endproducts; TAZ: Transcriptional Co-activator with PDZ-binding Motif; TEAD: TEA Domain; TGF-β: Transforming Growth Factor beta; TNF-α: Tumor Necrosis Factor α; TRP: Transient Receptor Potential; YAP: Yes-Associated Protein
